# Afghan women’s empowerment and antenatal care utilization: a population-based cross-sectional study

**DOI:** 10.1186/s12884-022-05328-0

**Published:** 2022-12-27

**Authors:** Sarah Yeo, Melanie Bell, Yu Ri Kim, Halimatou Alaofè

**Affiliations:** 1 Department of Health Promotion Sciences, Mel and Enid Zuckerman College of Public Health, University of Arizona, Tucson, AZ USA; 2 Department of Epidemiology and Biostatistics, Mel and Enid Zuckerman College of Public Health, University of Arizona, Tucson, AZ USA; 3 Asia-Pacific Research Center & School of International Studies, Hanyang University, Seoul, Republic of Korea

**Keywords:** Prenatal care, Antenatal care, Demographic and health survey, Maternal health, Women’s empowerment, Afghanistan

## Abstract

**Background:**

Although antenatal care (ANC) offers a unique opportunity to diagnose and prevent complications by mitigating modifiable risk, 38.2% of women did not complete any ANC visits in Afghanistan in 2015. Women empowerment is associated with increased use of ANC; however, there is no evidence of the effect of women empowerment on ANC in the country. Addressing this gap, we aimed to evaluate the association between women’s empowerment and ANC utilization based on the conceptual framework of women’s empowerment.

**Methods:**

We analyzed data from the 2015 Afghanistan Demographic and Health Survey for 11,056 women. The association between four domains of women’s empowerment, including capability, access to resources, security, and decision-making and power, and at least four ANC visits was analyzed using a multivariable logistic regression.

**Results:**

After adjusting for covariates, access to information (AOR 1.38, 95%CI 1.24, 1.54) and decision-making (AOR 1.16, 95%CI 1.08, 1.24) were positively associated with four or more ANC visits. Compared to those without any education, women with primary education (AOR 1.67, 95%CI 1.02, 2.72), secondary education (AOR 2.43, 95%CI 1.25, 4.70), and higher education (AOR 3.03, 95%CI 1.30, 7.07) had higher odds of least four ANC visits. However, asset ownership was negatively associated with ANC visits (AOR 0.72, 95%CI 0.56, 0.92). Variables related to security and literacy were not associated with the minimum ANC visits.

**Conclusions:**

The mixed results of the study highlight the complex natures of women’s empowerment, warranting a more nuanced understanding of women’s empowerment in the context and future research that capture multidimensionality of women’s empowerment. Also, efforts to empower women, particularly those with no education and had less decision-making power and access to health information, could be an effective strategy to enhance ANC use in Afghanistan.

## Background

Improving reproductive, maternal, neonatal, and child health outcomes remains a challenge in most of the developing world. In 2017 alone, approximately 295,000 women died from preventable causes associated with pregnancy and childbirth, and some parts of the world still bear a disproportionate burden of maternal deaths [1]. Afghanistan has 638 maternal deaths per 100,000 live births, making it one of the top ten countries with the highest maternal mortality ratio [1]. While the maternal mortality ratio declined by 30% globally from 1990 to 2015, Afghanistan, along with Sierra Leone and the Central African Republic, was one of the few countries with a worsening maternal mortality ratio [2].

Preventable causes such as antepartum hemorrhage, obstructed labor, pregnancy-induced hypertension, and sepsis claimed the lives of many women in the country [3], and antenatal care (ANC) offers a unique opportunity to diagnose and prevent such conditions by mitigating modifiable risks [4]. For this reason, the World Health Organization (WHO) has set a minimum threshold of four ANC visits, which is consistent with Afghanistan’s national guidelines [5]. In Afghanistan, however, less than 20% of women completed the recommended four or more ANC visits in 2015 [6]. While 48.1% of women in developing countries had early ANC visits, which are defined as visits done during the first trimester of pregnancy, women who completed early ANC visits were less than 30% in Afghanistan, with 38.2% of women having no ANC visits [6, 7].

An extensive body of literature investigated the factors that are associated with ANC utilization in developing countries in an attempt to promote ANC utilization and improve maternal health outcomes. In Afghanistan, women’s educational level, husband’s educational level, literacy, occupation, wealth status, residence, availability of owned transportation means were some of the factors that determine the level of service utilization [8, 9]. Recently, a growing body of literature started investigating the role of women’s empowerment on maternal health outcomes and maternal health care service utilization including access to ANC [10]. Several recent studies suggest that women’s empowerment and autonomy are positively associated with maternal health outcomes and maternal health care service utilization in developing countries [10–16].

The majority of research investigating the link between women’s empowerment and maternal health outcomes and use of maternal health care services has been in Sub-Saharan Africa or a few Asian countries like Bangladesh and India [10, 17]. To our knowledge, there has not been any study that investigated the association between women’s empowerment and ANC utilization in Afghanistan based on population-based survey data. Also, many studies lack theoretical frameworks or rationales that inform the selection of variables that construct the concept of women’s empowerment. We conducted this study to address the knowledge gap and contribute to the understanding of the association between women’s empowerment and the use of antenatal care services after adjusting for covariates using the conceptual framework that aligns with data available in the DHS.

## Methods

### Data source

The study analyzed data from the 2015 Afghanistan Demographic and Health Survey (2015 AfDHS). The DHS is a national cross-sectional survey that collects data on maternal and child health, family planning practices, nutritional status, mortality, fertility, knowledge and attitude concerning infectious diseases such as HIV/AIDS. As the first DHS conducted in Afghanistan, the survey collected data from a national sample of 25,650 households. Out of the surveyed households, ever-married women aged 15–49, who are members of the households and spent the night in the household before the survey, were included and interviewed (*n* = 29,461).

The survey is based on a two-stage stratified sampling with stratification of the 34 provinces into urban and rural areas using probability proportional to size selection at the first stage of sampling. The response rates for individual women surveys were high, with 97.5% in urban areas and 98.5% in rural areas. Data are included in this study if women were aged 15–49, had a live birth in the 5 years preceding the survey and their data were complete for all the variables used in the model. Responses such as “don’t know” were regarded as missing. The total number of observations used in the primary analysis was 11,056.

### Key variables and measurements

#### Women’s empowerment

To capture the intricate multidimensional nature of women’s empowerment, different variables from the DHS data have been used. However, combining and aggregating results from different indicators across different domains could preclude the possibility of a more nuanced understanding of women’s empowerment [18]. Therefore, this study used multiple variables related to women’s empowerment instead of aggregating them into an index or score.

The women’s empowerment variables are based on the four domains of gender equality and women’s empowerment suggested by Steward: capability, access to resources, security, and decision-making and power [19]. The capability domain covers women’s education, training, and health, while the access to resources domain includes assets and employment. The security domain includes protection from violence. Lastly, the domain of decision-making and power refers to political and economic participation and decision-making and control over financial resources [19].

**Fig. 1:**
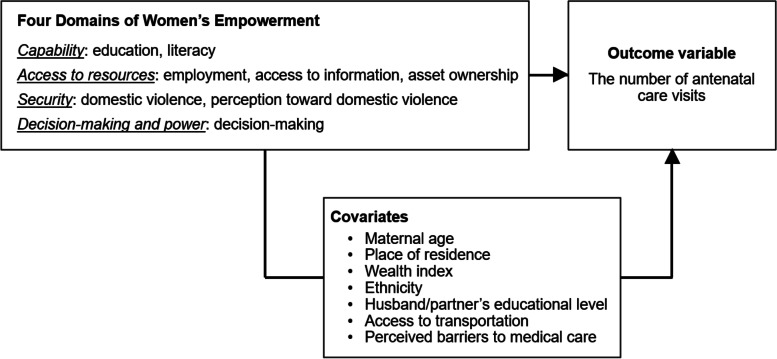
Conceptual framework of the association between women’s empowerment and antenatal care utilization based on the four domains of women’s empowerment

Figure 1 illustrates the conceptual framework of this study. Women’s education (no education, primary, secondary, higher) and literacy variables were included in the capability domain. Current working status, access to information (exposure to reading newspaper, listening to radio, or watching television, ranging 0–3 with 3 indicating exposure to all three media) and asset ownership (having ownership of house or land alone or jointly with husband/partner) fall in the domain of access to resources. The security domain included domestic physical violence (whether experienced any physical or sexual violence by husband/partner) and perception toward violence. To assess perception toward violence, participants were asked whether they agree with five different statements on situations when beating a wife is justified (ranging 0–5 with 5 indicating disagreement for all the five situations; if a wife argues with her husband, neglects the children, goes out without telling her husband, refuses to have sex with her husband, and burns the food). Finally, the domain of decision-making and power included a decision-making variable on whether the participants are involved in the decision-making process (alone or jointly) for their own health care, large household purchases, family or relative visits, and how to spend money earned by their husband (ranging 0–4 with 4 indicating respondents decide alone or jointly for all four situations).

#### Outcome variable

The primary outcome variable was ANC utilization measured by ANC coverage. ANC coverage is defined as “the percentage of women aged 15-49 with a live birth in a given time period that received ANC four or more times” based on the WHO guidelines [20]. Although the WHO’s revised guideline in 2016 recommends eight ANC contacts rather than four, the indicator is still in use because many countries are still failing to fulfill the minimum requirement of four ANC visits. Given that less than 5% of women completed the eight ANC visits in Afghanistan, we used the previous threshold. This variable dichotomized as to whether or not respondents attended four or more ANC visits.

#### Covariates

We identified potential confounders based on a body of literature on maternal health care utilization and its associated factors [8, 9, 11, 13, 21]. The variables include maternal age (categorized into 15–19, 20–29, 30–39, 40–49 in the model as it did not meet the assumption of linearity in the log-odds), partner/husband’s educational level (no education, primary, secondary, higher), region (urban/rural), ethnicity and wealth quintiles (the DHS wealth quintiles divide households into five categories: poorest, poorer, middle, richer, and richest based on household characteristics, assets, and access to goods and services.) [22]. Access to transportation (having either bicycle, motorcycle/scooter, or car/truck in the household) and any perceived barriers to medical care (whether respondents reported any perceived barriers such as getting money for treatment to get medical help for themselves) were also included in the adjusted model.

#### Statistical methods

Sampling weights were used for all the analyses and adjusted for the complex survey design. More details on how the sampling weights were calculated can be found elsewhere [6].

First, descriptive statistics were computed stratified on ANC visits (none, 1–3, ≥ 4). We reported frequencies, weighted proportions or means and standard errors were reported as appropriate. Then, using logistic regression models, unadjusted odds ratios and 95% confidence intervals were estimated to examine the association between each component and the receipt of four or more ANC visits. Finally, we fitted a multivariable logistic regression model after adjusting for maternal age, partner/husband’s educational level, region, ethnicity, wealth index, access to transportation, and perceived barriers to medical care.

Prior to the primary analysis, we checked the assumptions for logistic regression including linearity in the log-odds for continuous variables and identified influential observations using leverage, deviance residuals, and Pregibon’s Delta-Beta influence statistics. We did a sensitivity analysis by excluding the identified influential observations. We also checked collinearity based on the level of tolerance and variance inflation factor (VIF). All the analyses were conducted using Stata 16 (Stata Corp, College Station, TX).

## Results

A total of 11,056 women were included in the analysis, and Table 1 illustrates the characteristics of the study samples, which are organized by the number of ANC visits. 18.8% of women completed four times or greater ANC visits, whereas a majority of women (81.2%) completed less than four times visits. The women who completed ANC visits four times or greater were more likely to reside in urban areas and be wealthier and literate, and their husbands or partners tend to be better educated. The groups did not differ in terms of age or access to transportation. The total number of children was slightly fewer among the group that completed four or more ANC visits. A greater proportion of women reported barriers to medical care among women who completed less than four visits. A statistically significant difference was observed between different ethnic groups. Women that belong to Tajik and Hazara tend to have an increased number of ANC visits.

**Table 1:** Characteristics of study samples (women aged 15–49 in Afghanistan) by the number of antenatal care visits (weight adjusted) (*n* = 11,056)

	ANC visits < 4 (*n* = 9041, 81.2%)	4 ANC visits (*n* = 2015, 18.8%)	
	n (weighted %)^**1**^	n (weighted %)	***P***-value^**2**^
**Age** (mean, SE)	29.0 (0.1)	29.1 (0.3)	0.8
**Region**			< 0.001
Urban	1936 (65.5)	827 (34.5)	
Rural	7105 (85.8)	1188 (14.2)	
**Wealth Index**			< 0.001
Poorest	1875 (89.5)	242 (10.5)	
Poorer	2144 (88.4)	274 (11.6)	
Middle	2052 (85.3)	396 (14.7)	
Richer	1878 (79.0)	498 (21.0)	
Richest	1092 (63.4)	605 (36.6)	
**Ethnicity**			< 0.001
Pashtun	3822 (86.0)	553 (14.0)	
Tajik	2824 (74.6)	942 (25.4)	
Hazara	854 (77.4)	234 (22.6)	
Uzbek	535 (84.1)	172 (15.9)	
Others	1006 (88.1)	114 (11.9)	
**Husband/partner’s education level**			< 0.001
No education	5358 (85.0)	864 (15.0)	
Primary	1296 (83.1)	314 (16.9)	
Secondary	1899 (77.7)	553 (22.3)	
Higher	488 (56.0)	284 (44.1)	
**Access to transportation**^3^			0.8
No access	3997 (81.4)	861 (18.6)	
Access	5044 (81.1)	1154 (18.9)	
**Barriers to medical care**^4^			< 0.001
No reported barriers	646 (68.0)	348 (32.0)	
Any reported barriers	8395 (82.8)	1667 (17.2)	
**Total number of children**^5^ (mean, SE)	4.4 (0.1)	4.0 (0.1)	< 0.05

*Abbreviations*: *ANC* Antenatal care, *SE* Standard error

^1^Numbers and weighted row percentages

^2^Pearson’s chi-square test with correction for the complex design and survey weighted t-test (means) were used for comparison between the groups

^3^The percentage/number of those who have any means of transportation (bicycle, motorcycle/scooter, or car/truck) in the household compared to those who do not have any means

^4^Any perceived barriers to medical care (whether respondents reported any perceived barriers such as getting money for treatment to get medical help for themselves

^5^Total number of children ever born

In an unadjusted model, all the variables, except employment and domestic violence, were associated with higher odds of at least four ANC visits (Table 2). Among the variables, women’s education had the highest crude odds ratio. The crude odds ratios were 1.91 for primary education (95%CI 1.33, 2.73), 3.45 for secondary (95%CI 2.17, 5.49), and 5.77 (95%CI 2.36, 14.13) for higher education.

**Table 2:** Logistic regression odds ratios and 95% confidence intervals for the relationship between women’s empowerment and antenatal care utilization^a^ (*n* = 11,056)

	4 ANC visits (*n* = 2015,18.8%)	Unadjusted OR (95% CI)	***p***-value	Adjusted OR^b^ (95% CI)	***p***-value
**Capability**					
Education					
No Education	1407 (15.5)	1 (Reference category)		1 (Reference category)	
Primary	234 (26.0)	1.91 (1.33, 2.73)	< 0.001	1.67 (1.02, 2.72)	0.04
Secondary	268 (38.8)	3.45 (2.17, 5.49)	< 0.001	2.43 (1.25, 4.70)	0.01
Higher	106 (51.5)	5.77 (2.36, 14.13)	< 0.001	3.03 (1.30, 7.07)	0.01
Literacy	550 (34.4)	2.77 (1.89, 4.08)	< 0.001	0.66 (0.40, 1.09)	0.11
**Access to resources**					
Employment	182 (16.8)	0.86 (0.64, 1.15)	0.30	0.76 (0.57, 1.01)	0.06
Access to information^c^ (mean, SE)	1.22 (0.03)	1.83 (1.60, 2.09)	< 0.001	1.38 (1.24, 1.54)	< 0.001
Asset ownership^d^	791 (15.9)	0.72 (0.58, 0.90)	0.003	0.72 (0.56, 0.92)	0.01
**Security**					
Domestic violence^e^	824 (17.5)	0.83 (0.67, 1.03)	0.09	1.09 (0.87, 1.35)	0.45
Perception toward violence^f^ (mean, SE)	2.6 (0.1)	1.07 (1.02, 1.13)	0.01	1.00 (0.95, 1.06)	0.90
**Decision-making and power**					
Decision-making^g^ (mean, SE)	2.1 (0.1)	1.19 (1.11, 1.28)	< 0.001	1.16 (1.08, 1.24)	< 0.001

^a^Antenatal care utilization is defined by antenatal care visits ≥4

^b^Adjusted for maternal age, partner/husband’s educational level, region, ethnicity, wealth index, access to transportation, and perceived barriers to medical care

^c^The percentage/number of those who read newspaper, listen to radio, and watch television compared to those who do not

^d^Owns house or land alone or jointly

^e^Experienced any physical violence

^f^Scales ranging from 0 to 5 with greater numbers indicating more disagreement with itemized physical domestic violence

^g^Scales ranging from 0 to 4 with greater numbers indicating more decisions made alone or jointly

**Fig. 2:**
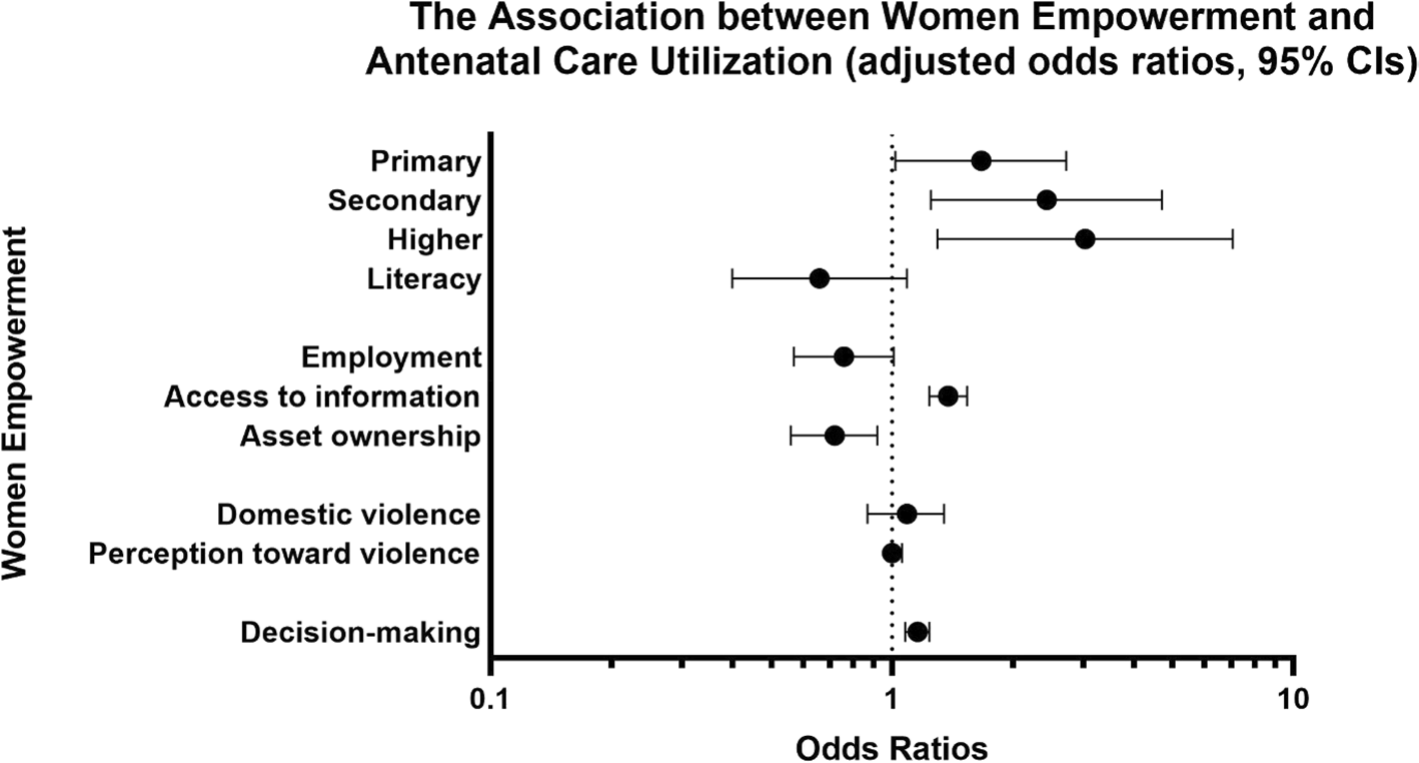
The odds ratios and CIs for the association between women’s empowerment and antenatal care utilization

After adjusting for maternal age, partner/husband’s educational level, region, ethnicity, wealth index, access to transportation, and perceived barriers to medical care, access to information and decision-making were associated with four or more ANC visits with an adjusted odds ratio (AOR) of 1.38 (95%CI 1.24, 1.54) and 1.16 (95%CI 1.08, 1.24), respectively (Table 2 and Fig. 2).

For the capability domain, compared to those without any education, women with primary education (AOR 1.67, 95%CI 1.02, 2.72), secondary education (AOR 2.43, 95%CI 1.25, 4.70), and higher education (AOR 3.03, 95%CI 1.30, 7.07) had greater odds of at least four ANC visits. One thing to note is literacy. The unadjusted odds ratio for literacy was 2.77 (95%CI 1.89, 4.08, yet after adjusting for covariables, the odds ratio was 0.66 (0.40, 1.09), although it was no longer statistically significant.

Under the adjusted model, none of the variables under the security domain were statistically significant. Other interesting results were employment and asset ownership in the domain of access to resources. Although access to information was still positively associated with greater odds of ANC visits (AOR 1.38, 95%CI 1.24, 1.54), employment and asset ownership were negatively associated with four or more ANC visits (AOR 0.76, 95%CI 0.57, 1.01 and AOR 0.72, 95%CI 0.56, 0.92, respectively) after adjusting. Decision-making was still positively associated with a greater odds of ANC visits (AOR 1.16, 95%CI 1.08, 1.24). Excluding influential observations, we performed a sensitivity analysis (*n* = 10,397) and overall, the results were consistent with the primary analysis, suggesting that the results are robust.

## Discussion

In this study, we investigated the association between women’s empowerment and ANC utilization in Afghanistan based on the theoretical framework of women’s empowerment. Overall, the results were mixed in our study: education, access to information, and decision-making were positively associated with four or more ANC visits.

Women’s higher education was significantly associated with adequate ANC care. The results of previous studies had shown a positive association with the level of education of women and ANC utilization [23, 24]. Our study also claimed that women’s decision making about their health care and autonomy to meet their friends or relatives was positively associated with ANC utilization, suggesting that high autonomy in women should be interpreted into improved health-seeking behavior and subsequently to generate better health outcomes [25]. Finally, women who had access to newspaper, radio, or television were more likely to use ANC as compared to women who had no access to these things. Various studies conducted in other developing countries showed that women’s exposure to media was positively associated with maternal health care utilization [12, 26, 27]. The media could be an effective way of information dissemination and enhance health care information and motivate women to utilize proper health care [28].

However, variables related to security and literacy were not associated with the minimum ANC visits after adjusting for covariates. Employment and asset ownership were negatively associated with ANC visits. Such mixed results are consistent with other studies to some degree. In one systematic review investigating the association between women’s empowerment and access to maternal care in developing countries, not all the indicators commonly used to measure women’s empowerment were significantly associated with health outcomes [10]. Part of this may be attributed to the multidimensional nature of women’s empowerment as noted in other relevant literature [10]. Women’s empowerment is multidimensional and a complex phenomenon, which is heavily influenced by culture, contexts, and time [18]. Thus, this convoluted nature necessitates a more nuanced understanding of each variable in the context of Afghanistan.

The negative effect of asset ownership, for example, could be explained by dowry culture in Afghanistan. Afghan brides are entitled to Mahr (dowry) which includes exclusive rights to land or property according to the country’s Civil Code [29]. Although dowry is one of the most common ways in which women acquire land and property rights, it is a cultural practice negotiated between the families, particularly among male elders [30]. Thus, having ownership and rights to land and house in the cultural contexts of Afghanistan may not simply translate into an indicator of women’s empowerment.

Looking further into the variables of employment also underscores the importance of a more nuanced understanding of women’s empowerment and its association with ANC visits for Afghan women. When the types of employment were considered, women who worked for someone else received more ANC visits than women who worked for family members or were self-employed [6]. Also, the types of occupations seemed to matter. When looking further into the data in the 2015 AfDHS, ANC visits were more common among women who engaged in services, clerical, and professional/technical jobs, as opposed to self-employed agriculture and unskilled manual labor. Finally, women paid in cash were more likely to have more ANC visits than those paid in-kind. The results are consistent with other studies which demonstrated that engaging in non-agricultural occupation was associated with births with skilled birth attendants [31]. Given the multifaceted nature of women’s empowerment, it is better suited to use and report each indicator separately rather than aggregating different indicators into a single index, as suggested by the literature [10].

The major limitation of this study is that the data fail to capture full dimensions of women’s empowerment particularly dimensions beyond individual levels. As noted by a number of authors, women’s empowerment is a process that entails changes at multiple levels and dimensions encompassing economic, socio-cultural, interpersonal, legal, political, and psychological spheres [10, 18, 32, 33]. Yet, in the study, the selection of variables has been constrained by what is readily available in the DHS data, and the variables mainly capture individual aspects of women’s empowerment. Furthermore, given the nature and availability of data, it is difficult to identify the pathways through which women’s empowerment influences antenatal care utilization. It may be possible that education could lead to a greater sense of self-efficacy or autonomy among women, ultimately resulting in better antenatal care utilization. Alternatively, there could be a more direct pathway. Women may have learned about the value of ANC, for example, through education or exposure to information.

Despite the limitations, the findings from this study make several contributions to the current literature. First of all, the study is based on the first population-based survey conducted in the country that covers most of the regions. Also, the study utilized a theoretical framework that informed the selection of variables concerning women’s empowerment.

### Conclusion

The mixed results of the study highlight the intricate natures of women’s empowerment. More research including qualitative research on the pathways would help contextualize the findings and capture complex nuances of women’s empowerment [34]. Future research would benefit from including factors that capture multidimensionality of women’s empowerment. Also, only a fraction of women utilized adequate antenatal care (≥ 4 ANC visits) according to the WHO recommendations. However, ANC utilization was more common among those who are educated, involved in household decision-making, and well-informed. Therefore, our findings suggest the need for policies and interventions that promote women’s education, empower women through awareness and information about maternal health care, and create an environment in which women can make their own decisions. Implementing those interventions may have the potential to ensure adequate antenatal care for Afghan women.

Also, although it may take more time to fully comprehend the ramifications of political turmoil in Afghanistan, the political changes at the macro level are likely to have cascading effects on the lives of individual women in the country. Thus, it would be critical to observe the trajectories of the changes: how the changes manifest themselves and affect women’s empowerment not only at the individual or relational levels but also at the macro level and how these changes lead to the lives of women in the country including maternal health outcomes and maternal health care utilization in the years to come.

## Data Availability

The datasets used for this study are publically available in the DHS program website after a registration process (https://dhsprogram.com/data/).
